# Supplementary material for: Phenotypic and functional features of B cells from two different human subcutaneous adipose depots
Source: PLoS One. 2023 Apr 27;18(4):e0285025. doi: 10.1371/journal.pone.0285025 (PMC10138852; doi:10.1371/journal.pone.0285025)
Supplement: Attachment — Submitted filename: Response to reviewers #2.docx [file pone.0285025.s001.docx]

Reviewer #1: The authors have made many edits in response to my initial review. Unfortunately, these changes did not resolve the questions to my satisfaction, many concerns remain. Here is a summary of my concerns.

Thank you very much! Below (in red) is our response to your additional comments. Changes in the second amended version of our manuscript are highlighted in yellow.

First, authors have added a statement in two different places in the Results and Introduction in this version which notes “…due to the ongoing process of class switch OCCURRING IN THE OBESE ADIPOSE TISSUE” (page 8) and “ongoing process of class switch occurring in the obese AT” (page 9). This is a very interesting point and one of great interest in the field. However, the paper the authors cite (their own) does not show this result, so this is an overstatement. The authors previously found CXCR5+ T cells and class-switched B cells are detectable in the SVF from the human adipose. This DOES NOT confirm that the class switching is happening in the adipose itself. This is a very important point, so it undermines the author’s credibility to over-interpret and over-state their own results. These statements need to be revised or removed.

Good point, thank you! We added a paragraph in the Introduction (we believe that it is better there than in Results and Discussion) to explain this point – we hope in a clarifying way for the reviewer and the readers.

In addition to the published data, we have evidence from 3 donors only of post-switch transcripts (Iμ-Cγ1) in B cells sorted from the SVF, measured by semiquantitative PCR. Therefore, we are confident that the obese AT is a site of ongoing class switch recombination.

There is a problem with the author’s solution to the reviewer concern in Figure 2. The authors have redrawn the gate in their top two panels to include only the CD21low cells as requested. This is now better. However, the actual percentages of cells provided for all the panels, including the 4 panels below which should have been sub-gated on the first panels are completely unchanged. This includes the percentages shown for all populations as well as the error ranges. It is not likely that altering the gate in the top panel to include fewer cells will leave all subsequent values and errors as EXACTLY the same. Please review your data analysis and update the presentation of the data to include only the cells in the newly drawn subgate. Given all the mistakes with the data so far, it will be essential to correct these flow plots and include a scatterplot of RNA expression in ALL 5 samples/treatment group plus the statistics so we can be reassured that this is a significant and meaningful change of Tbet expression in the CD21loCD95+ population across all the samples.

Another good point, thank you for pointing this to us in the first review. Please note that the frequencies of CD21lowCD95+ B cells were correctly calculated from the beginning (now checked again) even when the CD21 gate was not correct in the figure. I don’t know what happened with that gate when we first submitted Fig. 2. My apologies!

In Figure 3, again the authors have responded to the reviewer’s comments by reanalyzing the data and providing a new plot for the CD21 flow staining. The CD21 histogram now includes X-axis labels, which is helpful. However, the MFI of the isotype control sample is still almost double the MFI of the two experimental samples (Breast and pannus). This presents the same exact problem that existed with the first set of data- the isotype control provides a higher MFI than either of the experimental samples. This indicates that neither experimental sample has any positive expression of CD21 above background staining with an irrelevant antibody. It also means that these two samples cannot be compared to each other to make meaningful statements about their relative expression of “CD21” since they need to be considered negative for CD21 expression. Since this difference is the goal of the scatterplots, they need to be removed or the conclusion restricted to CD95 expression.

Autoimmune B cells are CD21low/negCD95+, therefore removing CD21 staining is not good if we want to show that these pathogenic B cells are expanded in the obese AT.

Unfortunately, the isotype control antibody for fluorochrome-conjugated CD21 (recommended by the vendor) has always given issues to us - but the MFI graph even with this isotype control antibody shows less CD21 expression in pannus as compared to breast samples. We have thawed the SVFs and re-stained them using another isotype control (a bit better than the previous one). A new Fig 3 is provided with this second amended submission.

It appears that Fig 6 is still labeled as 2-NBDG…I am guessing this means the previous label was correct, but in the first draft, the actual data inside the panel was inadvertently swapped with the ROS data in Fig 9?

Correct! Fig. 6 shows 2-NBDG results whereas Fig. 9 ROS results. The previous mistake has been fixed in the first amended version.

Labels and data presentation for Figure 9C looks corrected, but it would help to add a phrase in the results to state that the data in panel 9C includes data from both breast and pannus tissue, and it would be very helpful to identify which samples are breast vs pannus by using two colors or symbols in panel 9C. This should make the author’s conclusion even more apparent as the two groups should segregate differently.

This was not clarified because in Fig. 9C, as well as in every other figure of the paper, breast samples are identified with round symbols whereas pannus samples are identified with square symbols.

Reviewer #2: Author have address as their best all comments. There are some limitation that commonly occur when working with human samples that i understand cannot be fixed. For the most part, conclusions in the article are rigorously supported by data shown.

Thank you very much! We really appreciate this comment.
