# Supplementary material for: Phenotypic and functional features of B cells from two different human subcutaneous adipose depots
Source: PLoS One. 2023 Apr 27;18(4):e0285025. doi: 10.1371/journal.pone.0285025 (PMC10138852; doi:10.1371/journal.pone.0285025)
Supplement: Attachment — Submitted filename: Response to reviewers #3.docx [file pone.0285025.s002.docx]

Reviewer #2:

Dear reviewer, below (in red) is our response to your comments. Changes in the third amended version of our manuscript are highlighted in light blue.

1. Figure 8C shows ECAR measurements over time. Usually, when running mito stress kits for seahorse, when ECAR data is generated do not show the same pattern that OCR. It seems like Oligomycin (time point 20min) had no effect on ECAR, however, it is expected to increase ECAR levels, as cells tend to compensate the inhibition of ATP produced by the mitochondria. Additionally, FCCP and Rot/AA do not have an effect in ECAR. Because FCCP promotes maximal respiration, that should not reduce acidification rate. Another thing worth checking is the values on the Y axis for ECAR. Normally, they are (at least the basal) within the range of the OCR value. I encourage authors to review raw data to make sure the data shown is correct or explain why this might have happened.

We have checked our ECAR results as well as results from other groups in the literature. We can say that ECAR profiles may show either a small effect of oligomycin (if any) as well as a reduction of ECAR values after injection of Rot/AA (as an example, please see refs from Dequina et al. (DOI: [10.1016/j.cmet.2019.07.004](https://doi.org/10.1016/j.cmet.2019.07.004) and DOI: [10.1371/journal.pone.0170975](https://doi.org/10.1371/journal.pone.0170975)).

To further support our results in Fig. 8, we would like to tell the reviewer that we have also run glycolytic tests in Seahorse injecting Glucose, oligomycin and 2-DG, and these tests have confirmed a highly glycolytic phenotype of B cells from the pannus as compared to those from the breast. Please see these results below (pannus=squared symbols, breast=round symbols).

2. When ROS were measured, were cells fixed? I was concerned about the incubation times as usually ROS production in live cells is measured within 20-30min. As it is described in material and methods, ROS were measured ~1h after the dye was added. If authors have some kind of positive control for ROS, like H2O2 treatment will help clarifying this.

As to the first point, cells were not fixed, they were acquired immediately at the end of staining (as already indicated in Materials and methods, paragraph “Detection of Reactive Oxygen Species (ROS) using CellROX Oxidative Stress Reagent”).
When referring to ROS, I suggest using the word "production" rather than "ROS expression".

Thank you for the suggestion. We changed the text accordingly (pages 13-14).

3. Figure 7 shows increased LDH expression in pannus samples. Authors suggest that this increase is due to pannus being more hypoxic than breast environment. This statement is an overinterpretation, since usually in vitro culture conditions do not mimic hypoxic conditions in the body and also because PDH mRNA levels are also upregulated. I would suggest a more detailed and careful explanation/correlation. Moreover, the fact that both, OCR and ECAR are higher in pannus cells, will implicate that these cells are more metabolically active than cells from breast tissue, but does not necessarily mean these cells are more glycolytic and/or anaerobic. Authors should extend the discussion on why these changes in metabolism could be occurring once ECAR data is reviewed.

Thank you also for this suggestion. We changed the text accordingly (pages 12-13).
